# Supplementary material for: Identification of photoperiod-induced specific miRNAs in the adrenal glands of Sunite sheep (Ovis aries)
Source: Front Vet Sci. 2022 Jul 22;9:888207. doi: 10.3389/fvets.2022.888207 (PMC9354845; doi:10.3389/fvets.2022.888207)
Supplement: Supplementary file 1 [file Data_Sheet_1.ZIP › Supplementary file/miRDeep2 file.zip!/miRDeep2 file/Novel_Structure/SPLP42A1/Novel_31.pdf]

Provisional ID	:	NW_014639514.1_32865
Score total	:	115.5
Score for star read(s)	:	3.9
Score for read counts	:	107.7
Score for mfe	:	2.3
Score for randfold	:	1.6
Score for cons. seed	:	
Total read count	:	223
Mature read count	:	215
Loop read count	:	0
Star read count	:	8

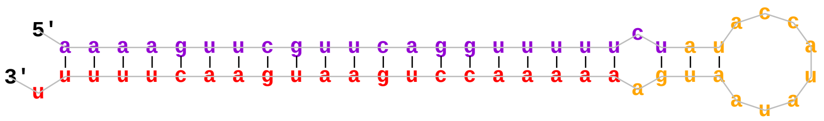

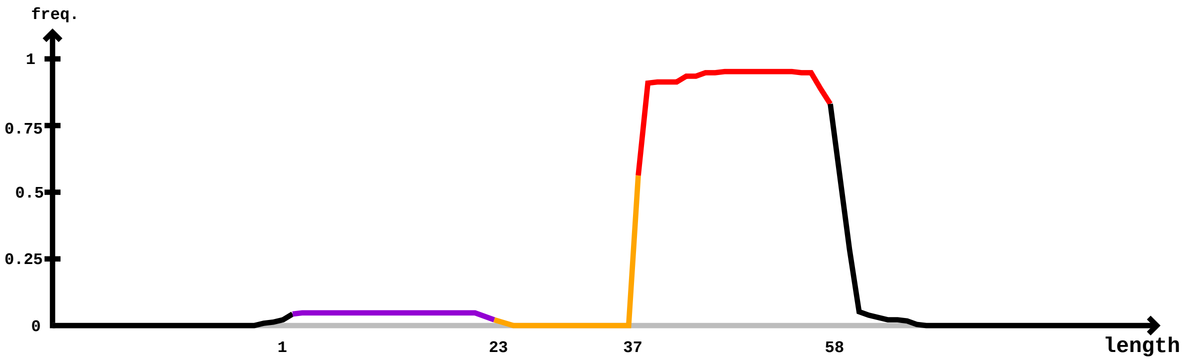

Star Mature

```
5'- uuccauaaauacuggguugggcaaaaaguucguucagguuuuucuauaccauauaaugaaaaaaccugaaugaacuuuuuggccaacccuguauuuuccuugguggcugcaaa   -3'   obs
    uuccauaaauacuggguugggcaaaaaguucguucagguuuuucuauaccauauaaugaaaaaaccugaaugaacuuuuuggccaacccuguauuuuccuugguggcugcaaa         exp
    ..((((((((((.(((((((.((((((((((((((((((((((.(((........))).)))))))))))))))))))))).))))))).))))))......)))).......   reads   mm   sample
    ....................gcaaaaaguucguucagguuuuu......................................................................   2   0   seq
    .....................caaaaaguucguucGgguuuuu......................................................................   1   1   seq
    ......................aaaaaguucguucGgguuuuuc.....................................................................   2   1   seq
    .......................aaaaguucguucGgguuuuucu....................................................................   5   1   seq
    ........................aaaguucguucaggCuuuuc.....................................................................   1   1   seq
    ...........................................................aaaaaccugaaugaacC.....................................   1   1   seq
    ...........................................................aaaaaccugaaugaacuuu...................................   1   0   seq
    ...........................................................aaaaaUcugaaugaacuuu...................................   9   1   seq
    ...........................................................aaaaaccugaaGgaacuuu...................................   1   1   seq
    ...........................................................aaaaaccAgaaugaacuuu...................................   1   1   seq
    ...........................................................Gaaaaccugaaugaacuuuu..................................   1   1   seq
    ...........................................................aaaaaccugaaugaacuuuu..................................   1   0   seq
    ...........................................................aGaaaccugaaugaacuuuu..................................   1   1   seq
    ...........................................................aaaaaccAgaaugaacuuuu..................................   7   1   seq
    ...........................................................aaaaaUcugaaugaacuuuuu.................................   80  1   seq
    ...........................................................Caaaaccugaaugaacuuuuu.................................   1   1   seq
    ...........................................................aaaaaccugGaugaacuuuuu.................................   1   1   seq
    ...........................................................aaaaaccugaaugaacuuuuC.................................   2   1   seq
    ...........................................................Gaaaaccugaaugaacuuuuu.................................   1   1   seq
    ...........................................................aaaaaccugaGugaacuuuuu.................................   1   1   seq
    ...........................................................aaaaaccAgaaugaacuuuuu.................................   1   1   seq
    ...........................................................aaaaaccAgaaugaacuuuuug................................   3   1   seq
    ...........................................................Gaaaaccugaaugaacuuuuug................................   1   1   seq
    ...........................................................aaaaaccugaaugaacCuuuug................................   1   1   seq
    ...........................................................aaaaaccugaaugaacuuuuuA................................   4   1   seq
    ...........................................................aaaaaccugaaGgaacuuuuug................................   8   1   seq
    ...........................................................aaaaaccugaaugaacuuuuugU...............................   2   1   seq
    ...........................................................aaaaaccugaaugaacuuuuuUg...............................   1   1   seq
    ............................................................aaaaccAgaaugaacuuu...................................   1   1   seq
    ............................................................aaaaccugaaugaacuuu...................................   1   0   seq
    ............................................................aaaaccAgaaugaacuuuu..................................   1   1   seq
    ............................................................aaaaccugaaugaacuuuu..................................   1   0   seq
    ............................................................aaaaccugGaugaacuuuu..................................   1   1   seq
```

```
                       Star                                  Mature
uuccauaaauacuggguugggcaaaaaguucguucagguuuuucuauaccauauaaugaaaaaaccugaaugaacuuuuuggccaacccuguauuuuccuugguggcugcaaa
............................................................Gaaaccugaaugaacuuuuu.................................    1    1    seq
............................................................aaaaccugaaugaacuuuuu.................................    11   0    seq
............................................................aaaaccugaaugaacCuuuu.................................    1    1    seq
............................................................aaaaccGgaaugaacuuuuu.................................    1    1    seq
............................................................aaaaccAgaaugaacuuuuu.................................    3    1    seq
............................................................aaaaccugaaugaacuuuuuA................................    1    1    seq
............................................................aaaaccugaaugaacCuuuug................................    3    1    seq
............................................................aaaaccugaaugaacuuuuugg...............................    34   0    seq
............................................................aaaaccugaaugaacCuuuugg...............................    5    1    seq
............................................................aaaaAcugaaugaacuuuuugg...............................    1    1    seq
............................................................aaaaccugaaugaacuuuuugU...............................    1    1    seq
............................................................aaaaccugaaugaacuuuuugA...............................    10   1    seq
............................................................aaaaccugaaugaacuuuuuggU..............................    1    1    seq
............................................................aaaaUcugaaugaacuuuuuggc..............................    2    1    seq
.............................................................aaaccGgaaugaacuuuuug................................    1    1    seq
................................................................ccugaaugaacuuuuuggcc.............................    2    0    seq
................................................................ccugaaugaacuuuuuggccC............................    1    1    seq
................................................................ccugaaugaacuuuuuggcca............................    1    0    seq
................................................................ccugaaugaacuuuuuggccaUc..........................    1    1    seq
..................................................................ugaaugaacuuuuuggccaacc.........................    1    0    seq
..................................................................ugaaugaacuuuuuggccaacA.........................    1    1    seq
..................................................................ugaaugaacuuuuuggUcaaccc........................    1    1    seq
....................................................................aaugaacuuuuuggccaacc.........................    1    0    seq
```
